# Experimental Investigation of Dispersant on Dynamics of Impact of Al_2_O_3_ Nanofluid Droplet

**DOI:** 10.3390/nano15020108

**Published:** 2025-01-12

**Authors:** Dandan Liang, Ruichao Guo, Zichun Sun, Haizhen Zhao, Guohua Qin, Yongxin Zhang

**Affiliations:** 1 School of Aeronautical Engineering, Shandong Engineering Research Center of Aeronautical Materials and Devices, Shandong University of Aeronautics, Binzhou 256600, China; 2 School of Aeronautical Manufacturing Engineering, Nanchang Hangkong University, Nanchang 330063, China

**Keywords:** nanofluid, droplet impact, dispersant, spreading factor, weber number

## Abstract

Spray cooling, of which the essence is droplet impacting, is an efficient thermal management technique for dense electronic components in unmanned aerial vehicles (UAVs). Nanofluids are pointed as promising cooling dispersions. Since the nanofluids are unstable, a dispersant could be added to the fluid. However, the added dispersant may influence the droplet, thereby impacting behaviors. In this work, the effects of dispersant on the nanofluid droplet-impacting dynamics are studied experimentally. The base fluid is deionized water (DI water), and Al_2_O_3_ is the selected nanoparticle. Sodium dodecyl sulfate (SDS) is used as the dispersant. Five different concentrations of nanofluids are configured using a two-step method. Droplet impacting behaviors are observed by high-speed imaging techniques. The other effects, i.e., the nanofluid particle volume fraction and the Weber number on droplet impact dynamics, are also systematically investigated. The results illustrate that the surface tension of the Al_2_O_3_ nanofluid increases with increased nanofluid concentrations. The surface tension of Al_2_O_3_ nanofluid with SDS is lower than that of DI water. And the increase in droplet impact velocity increases the spreading morphology. Nanofluid droplets exhibit spreading and equilibrium process when SDS is added. Furthermore, as the concentration of the nanofluid increases, the spreading process is inhibited. Whereas without SDS, the droplets undergo spreading, receding, and equilibrium processes. Moreover, there is no appreciable change in the impacting process with concentration increase. The empirical models of maximum spreading factor should be established without SDS and with SDS, respectively. This study can provide theoretical basis and specific guidance for experimental characterization of UAVs’ electronic devices based on the mechanism of nanofluid droplet impact on the wall.

## 1. Introduction

Unmanned aerial vehicles (UAVs) are emerging equipment that have developed rapidly in recent years and have been widely used in many fields, e.g., military reconnaissance [1], pesticide spraying [2], and disaster management [3]. The UAVs, with high scientific and technological content and industrial added value, are leading the trend of innovation in modern technology and application fields. With the continuous upgrading and iteration of drone technology, the number and types of electronic devices of the UAVs have shown an alarming growth trend. These electronic devices not only have different functions but are also becoming increasingly miniaturized, resulting in a geometric leap in the density of electronic devices inside the UAVs. However, the dramatic increase in the density of electronic devices has also brought about a series of problems such as heat dissipation [4,5].

The traditional cooling methods of the electronic components inside the UAVs include radiator cooling, fan cooling, and air cooling. However, the traditional cooling methods can no longer meet the design requirements for high power and high heat flow density equipment. Thus, the innovation and optimization of cooling technology is still a bottleneck in the development process of UAVs. And it is imperative to seek an efficient and fast cooling method. Spray cooling, which is a process of finely divided droplets hitting the wall, provides a very effective solution [6]. However, the present changes in process parameters in spray cooling have limited improvement in heat flow density. Further search for efficient cooling media is still needed [7].

Nanofluid is a new type of energy transporting medium with high efficiency and high heat transfer performance. The nanofluid is generally regarded as the next generation of coolant due to its advantages of high thermal conductivity, large specific surface area, micro-convection, and lower corrosion ability [8]. Buschmann et al. [9] found that the heat transfer enhancement provided by nanofluids equaled the increase in the thermal conductivity of the nanofluid. Aksoy et al. [10] found that there was a heat transfer increase by the use of nanofluids, but high particle concentrations could affect nozzle clogging and heat transfer potential could be limited. However, Eneren et al. [11] showed that there was no remarkable heat transfer enhancement in nanofluids at different concentrations.

In addition to spray cooling, the physical mechanism of droplet impact on solid surfaces is widely used in engineering fields such as pesticide spraying, inkjet printing [12,13], engine fuel inhalation, and steam condensation in turbines [14,15]. The dynamic behaviors of a liquid droplet impacting on a solid surface show complex phenomena such as spreading, rebounding, splashing, and breaking [16,17]. A large number of studies have shown that the kinetic behavior of the droplet impacting on the wall is related to the solid surface state, the nature of the liquid thermophore, and the state of the droplet, becoming a hotspot and a difficult point of research.

As early as 1877, Worthington [18] carried out the first experimental study of liquid droplets striking a solid surface. Zhang et al. [19] experimentally investigated the effect of surfactant concentration on the rebound and spreading of a droplet during impact on a solid wall. They conducted that the surface tension of droplets decreased with the increase in surfactant concentration, which lead to the enhancement of droplet spreading. An et al. [20] investigated the process of shear-thinning fluid droplets impacting on surfaces with different wettability. The viscosity of the droplets was found to change during the impact process and ultimately affected the spreading of the droplets.

Nanofluids are suspensions formed by dispersing nanoparticles such as metals into the base fluid. The addition of nanoparticles alters the thermal properties of the base fluid and significantly affects the dynamic behavior of droplet impact on surfaces. Zang et al. [21] investigated the impact of SiO_2_ nanofluid droplets on surfaces with and without polyethylene glycol additives. They found that the addition of additives increased friction between nanoparticles and polymer aggregates, suppressing droplet rebound during high-speed impacts. Hao et al. [22] prepared a SiO_2_ nanofluid using ethylene glycol as the base fluid. They noted that increasing fluid concentration inhibited nanofluid droplet rebounding phenomena. Li et al. [23] prepared a TiO_2_ nanofluid. They discovered that adding a small amount of hydrophilic TiO_2_ nanoparticles effectively suppressed droplet rebounding on solid surfaces. Li et al. [24] found that dodecyl dimethyl ammonium bromide, a double-chain cationic surfactant, could inhibit droplets from receding and rebounding at an ultralow concentration (0.05%). Esmaeili et al. [25] explored the key role of molecular weight and concentration in the droplet impact dynamics of nonionic surfactant solutions. Thoraval et al. [26] found that the splashing of a nanoparticle dispersion could be suppressed at higher impact velocities by the interactions of the nanoparticles with the solid surface. Aksoy et al. [27] found that even a small amount of added nanoparticles had an impact on the splashing transition.

In summary, the present studies have focused on the impact of nanofluid droplets on a wall in a single case scenario. Due to strong van der Waals forces between nanoparticles, agglomeration is common in nanofluids. To mitigate this, dispersants like surfactants are often added to enhance nanofluid stability [28]. However, there also need nanofluids without dispersant in many cases [29,30,31]. Dispersant addition alters fluid thermophysical properties such as surface tension, thereby influencing the droplet spreading process. To the authors’ knowledge, there is no prior work on nanofluids to describe the dispersant changes in the droplet-impacting behaviors. Hence, further investigation into the impact of dispersant on droplet spreading is warranted.

In the present work, the preparation and characterization of a water-based Al_2_O_3_ nanofluid, without or with a dispersant, have been performed. Varied volume fractions of nanofluids are prepared by the two-step method. Subsequently, based on high-speed image technology, the process of nanofluid impingement on the wall is visualized for different conditions. And then, the study analyzes the Weber number, nanofluid concentration, and the absence or presence of dispersant on the dynamic parameters (spreading factor) of droplet impact. Finally, a predictive model for maximum spreading factor is proposed. We believe that this study shall provide a theoretical basis and specific guidance for experimental characterization of UAV electronic devices based on the mechanism of nanofluid droplet impact on wall surface.

## 2. Experimental Methods

### 2.1. Experimental Setup

**Figure 1:**
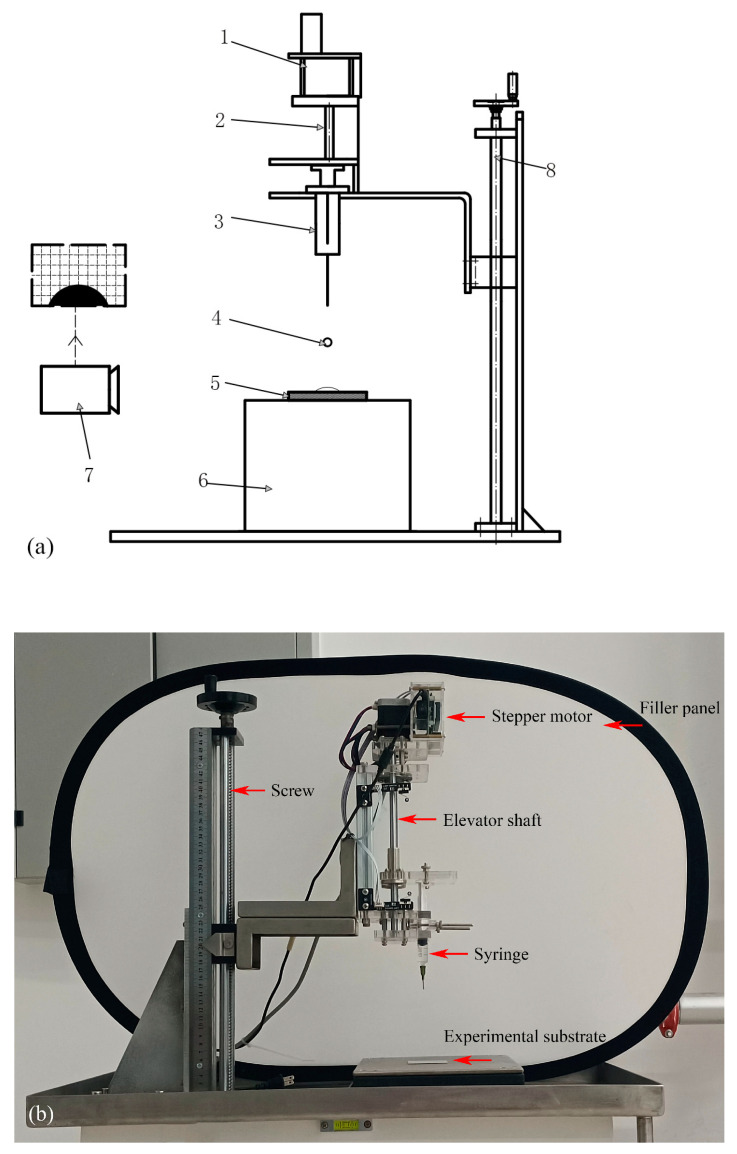
(**a**) Schematic and (**b**) Experimental system for droplet impact on wall surface. 1 Stepper motor; 2 Elevator shaft; 3 Syringe; 4 Droplet; 5 Experimental substrate; 6 Test bench; 7 High speed camera; 8 Screw.

A schematic illustration and an actual photograph of the experimental setup are shown in Figure 1. Basically, the apparatus consists of two components: the droplet generation system and the image capture system. In the droplet generation system, a stepper motor precisely controls the frequency of droplet generation through the elevation axis. The droplets of different diameters are generated by the changed caliber syringes (used one caliber syringe in this work). The droplet falling height is adjusted by the screw, and the droplet impact object in present work is a 7050 aluminum alloy plate. All impact objects have a uniform dimension of 50×50×0.5 mm, and each aluminum surface is prepared with surface roughness value of 0.8 μm (Rz). The surface is cleaned using high concentration alcohol to ensure the surface is absent of any impurities.

In the image capture system, a high-speed camera (Phantom VEO 410, Dantec Dynamcis, Wayne, NY, USA) with a macro lens is used to capture the droplet impact process. A 100 W LED light is used to provide back lighting for the dynamics of object droplets. Moreover, to obtain high quality photography, a Tokina^®^ (Tokyo, Japan) 12.5–75 mm micro lens is used to the camera. In this experiment, the aperture, the image resolution, the frame rate, and the exposure time are selected as 2.8, 1280 × 800, 4000 fps and 100 ms, respectively.

### 2.2. Nanofluid Preparation

Currently, the main preparation methods for nanofluids are one-step and two-step. The one-step method is the production of nanoparticles in one step, which avoids the storage, transportation, and dispersion of nanoparticles. However, this method has high cost, low yield, and is not suitable for industrial production. The two-step method divides the preparation of nanoparticles and the dispersion of nanoparticles into two steps. In the two-step method, the nanoparticles are obtained first and then dispersed into the liquid, showing simple operation and wide applicability. Therefore, the two-step method is chosen for the preparation of the nanofluid in this experiment.

The nanoparticles used in this work are aluminum trioxide particles (Al_2_O_3_, Nanjing Xianfeng Nanomaterials Technology Co., Ltd., Nanjing, China). The nominal diameter and the purity of Al_2_O_3_ is about 10–15 nm and 99.9 wt %, respectively. The Al_2_O_3_ nanoparticles are chosen for investigation, owing to their high heat transfer capability, followed by the dynamics of nanofluid droplets impingement on headed wall in further research [32]. In addition, the stabilization is very important for nanofluid preparation, which can be achieved by adding dispersants and ultrasonic vibration [28]. The dispersant used in the experiment is SDS. SDS, which is a kind of anionic surfactant, is one of the most used stabilization dispersants for Al_2_O_3_ nanofluid preparation [33]. The surfactant SDS produced by Sinopharm (Shanghai, China) is added as a dispersant with a purity of 99.0%. DI water is selected as the base solution. The nanofluids without SDS (NFNS) and with SDS (NFWS) are prepared, respectively.

To configure the nanofluids, the base solution, dispersant, and nanoparticles are added sequentially. The dispersions are stirred with a glass rod evenly, followed by electric machinery (JJ-1 60W, Changzhou Surui Instrument Co., Ltd., Changzhou, China) stirred for 10 min. Finally, the dispersions are ultrasonicated (KQ-300DE, Kunshan Ultrasonic Instrument Co., Ltd., Kunshan, China) at 28 kHz for 3 h. To prevent the nanofluid from overheating, the ultrasound is stopped every 40 min for 3 min. After ultrasonic dispersion, the nanofluid is allowed to stand for 0.5 h and cooled to room temperature. It should be noted that SDS is added with a uniform mass fraction of 0.25% [34,35]. In the present work, nanoparticle suspensions with volume fractions of 0.1–0.5% are configured. The volume fractions are calculated using the following equation [36]:

(1) $$\left\{\begin{array}{l} \phi =\frac{V_{Al_{2}O_{3}}}{V_{Al_{2}O_{3}}+V_{water}}=\frac{w_{Al_{2}O_{3}}/\rho_{Al_{2}O_{3}}}{w_{Al_{2}O_{3}}/\rho_{Al_{2}O_{3}}+w_{water}/\rho_{water}}\;NFNS\\ \phi =\frac{V_{Al_{2}O_{3}}}{V_{Al_{2}O_{3}}+V_{water}+V_{SDS}}=\frac{w_{Al_{2}O_{3}}/\rho_{Al_{2}O_{3}}}{w_{Al_{2}O_{3}}/\rho_{Al_{2}O_{3}}+w_{water}/\rho_{water}+w_{SDS}/\rho_{SDS}}\;NFWS \end{array}\right.$$

where $w_{Al_{2}O_{3}}$ is the weight of nanoparticles, $w_{water}$ is the weight of base solution, $\rho_{Al_{2}O_{3}}$ is the density of nanoparticles, and $\rho_{water}$ is the density of base solution.

**Figure 2:**
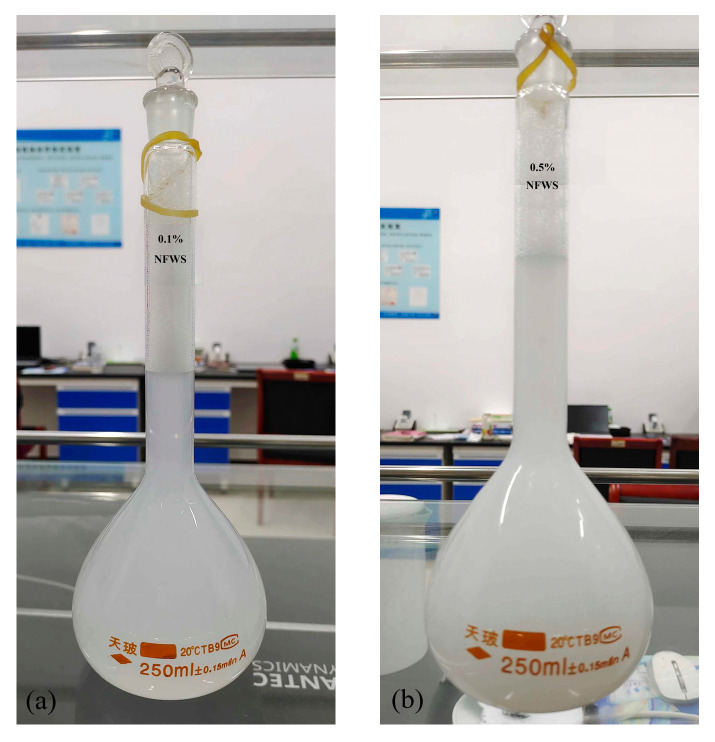
The prepared nanofluids. (**a**): 0.1%, NFWS and (**b**): 0.5%, NFWS.

The main experimental material parameters of this paper are shown in Table 1. The configured Al_2_O_3_ nanofluids with different concentrations are shown in Figure 2.

### 2.3. Nanofluid and Droplet Characterization

The surface tension tester (XR-6541) is used to measure the surface tension value of 0.1–0.5% gradient volume fraction of Al_2_O_3_ nanofluid by using the ring method. The surface tension of the liquid is indirectly determined by measuring the maximum pull required to pull the ring from the surface of the liquid [37]. The average value is measured three times for each group of tests in order to keep the statistical accuracy. The density of the nanofluid is calculated by the following equation [38]:

(2) $$\left\{\begin{array}{l} \rho_{f}=\phi_{Al_{2}O_{3}}\cdot \rho_{Al_{2}O_{3}}+\left(1-\phi_{Al_{2}O_{3}}\right)\cdot \rho_{water}\;NFNS\\ \rho_{f}=\phi_{Al_{2}O_{3}}\cdot \rho_{Al_{2}O_{3}}+\phi_{SDS}\cdot \rho_{SDS}+\left(1-\phi_{Al_{2}O_{3}}-\phi_{SDS}\right)\cdot \rho_{water}\;NFWS \end{array}\right.$$

where ϕ is the volume fraction of nanofluids.

The variation in surface tension and density at different concentrations are shown in Table 2. With SDS added, it can be seen that there is no obvious changes for density, but the surface tension changes dramatically.

In the absence of SDS, the value of surface tension increases slightly when the concentration of nanofluids changes from 0.1% to 0.5%. The value of surface tension is about 72.58 mN/m at 0.5% volume fraction. While in the presence of SDS, the surface tension values basically remain around 37.5 mN/m. And the change in the concentration of nanofluid does not have much effect on the value of surface tension.

For nanofluids, the interaction forces between the nanoparticles and the base fluid molecules are primarily a mutual attraction. On the one hand, as the number of nanoparticles increases, the attraction between the molecules increases, and the distance between the molecules decreases [39]. Therefore, the work required to overcome the cohesive forces, known as the surface tension, will increase.

On the other hand, when SDS is added, there forms a barrier layer between the particles and the surrounding fluid molecules. The formed layer increases the electrical potential between the particles and the created repulsive forces, leading to the decrease in surface tension. In conclusion, the surface tension increases with the increase in nanoparticle content, while the surface tension decreases with the addition of surfactant.

In the experiment, different impact velocities are obtained by adjusting the distance between the droplet and the impacting aluminum plate. With the obtained distance between the consecutive image in the vertical direction, the impact velocity can be calculated by the following equation:

(3) $$v_{d}=\frac{y_{2}-y_{1}}{\Delta t}$$

where $y_{2}$ is the position of droplet before impacting, $y_{1}$ is the position of droplet impacting the wall, and Δt is the frame rate.

**Figure 3:**
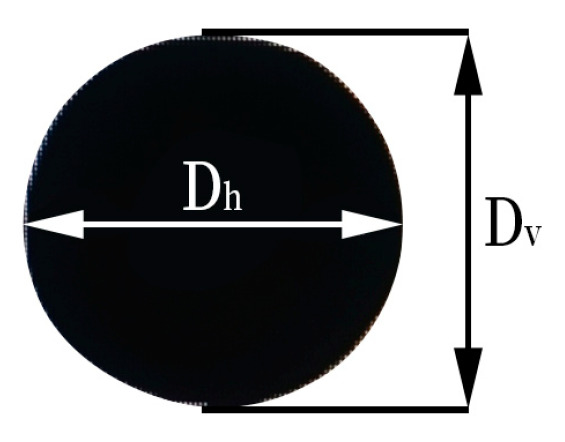
Definition diagram of droplet length of horizontal direction and vertical direction.

It is worth noting that the droplet does not assume a standard spherical form during the falling process. Under the influence of gravity, the droplet, in which the length in the vertical direction is larger than that in the horizontal direction, is an ellipsoid. The diameter of initial droplet diameter can be calculated by [40]

(4) $$D_{0}={\left(D_{v}D_{h}^{2}\right)}^{1/3}$$

where $D_{h}$ is the length of the droplet in the horizontal direction, and $D_{v}$ is the length of the droplet in the vertical direction. Using the ImageJ software (V1.51j8), the binarized image for the droplet is shown in Figure 3.

The most important non-dimension related to droplet impact is the We number, which is the ratio of droplet kinetic energy to surface tension, defined as

(5) $$We=\frac{\rho_{f}v_{d}^{2}D_{0}}{\sigma }$$

where $\rho_{f}$ is the density of the droplet, $v_{d}$ is the impact velocity of the droplet, $D_{0}$ is the initial diameter of the droplet, and σ is the surface tension. The impact conditions of nanofluid droplets are given in Table 3.

The dimensionless parameter of spreading factor is defined as [16,41,42]

(6) $$\beta =\frac{D\left(t\right)}{D_{0}}$$

where $D\left(t\right)$ is the time evolution of spreading diameter.

### 2.4. Uncertainty Analysis

Experimental errors inherent in the measurement of the independent variables lead to uncertainty in the calculated parameters. The height of the dropper is based on a ball screw unit with calipers, which has an accuracy of ±0.1 mm. The estimated maximum error in the droplet shape is about 1 pixel (equivalent to 0.1 mm with a 0.1 mm/pixel spatial resolution). The weight uncertainty of the surfactant and nanoparticles is ±0.1 g based on the performance of the precision balance. And the surface tension is obtained by Hill’s XR-6541 Surface Tension Tester. The accuracy of the tester is ±0.1 mN/m. The contact angle of the droplet is measured by a contact angle measuring instrument (SDC-100SY) with an accuracy of $\pm 1^{\circ }$. The error of $D_{0}$, $\beta_{\max}$, and Weber number can be calculated by the following equation [43]:

(7) $$\left\{\begin{array}{l} \frac{\delta D_{0}}{D_{0}}=\sqrt{{\left(\frac{1}{3}\frac{\partial D_{0}}{\partial D_{v}}D_{v}\right)}^{2}+{\left(\frac{2}{3}\frac{\partial D_{0}}{\partial D_{h}}D_{h}\right)}^{2}}\\ \frac{\delta \beta_{\max}}{\beta_{\max}}=\sqrt{{\left(\frac{\partial \beta_{\max}}{\partial D_{\max}}D_{\max}\right)}^{2}+{\left(\frac{\partial \beta_{\max}}{\partial D_{0}}D_{0}\right)}^{2}}\\ \frac{\delta W_{e}}{W_{e}}=\sqrt{{\left(\frac{\partial W_{e}}{\partial D_{0}}D_{0}\right)}^{2}+{\left(2\frac{\partial W_{e}}{\partial v_{d}}v_{d}\right)}^{2}+{\left(\frac{\partial W_{e}}{\partial \sigma }\sigma \right)}^{2}} \end{array}\right.$$

The main errors of the actual and measured values are listed in Table 4. The calculated uncertainty of the Weber number in this paper is ±7.38%.

## 3. Results and Discussion

### 3.1. Analysis of the Spreading Pattern of the Impact Process

**Figure 4:**
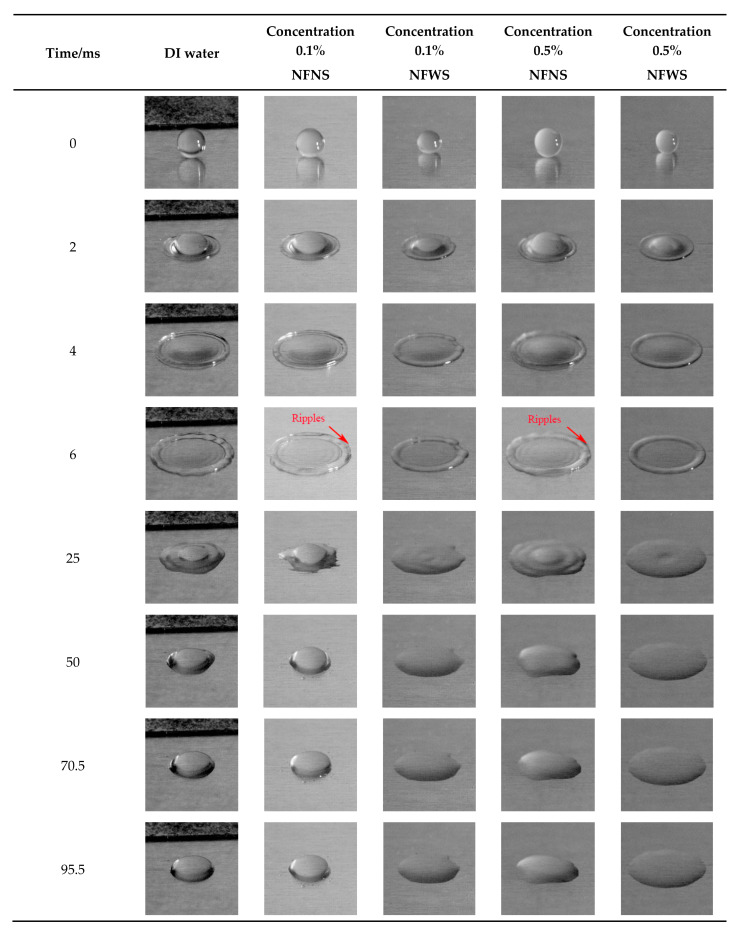
Impact of liquid droplets in different states on the wall of a 7050 aluminum alloy at 1.13 m/s.

When the impacting velocity is 1.13 m/s, Figure 4 shows the impacting behaviors of NFNS and NFWS droplets. Three different concentrations, which are 0% (DI water), 0.1%, and 0.5%, are compared in Figure 4.

It can be seen that the deformation process of the liquid droplet impacting the 7050 wall can be generally divided into three processes: spreading, receding, and equilibrium, with no splash phenomenon occurring. For DI water, at 6 ms after the droplet impacts the wall, the spreading process is observed. After the droplet spreads to its maximum diameter, it enters the receding process under the action of surface tension. Finally, the droplet reaches the equilibrium at the end of droplet receding (*t* = 50 ms).

Similarly, for both 0.1% and 0.5% NFWS nanofluids, it can be observed that the maximum spreading occurs at 6 ms when the droplet impacts on the wall. Furthermore, the droplet remains stable after 6 ms. In comparison, the maximum spreading is also achieved at 6 ms for both 0.1% and 0.5% NFNS nanofluids. However, it is observed to be in the receding process between 6.5 ms and 70 ms. Subsequently, it stabilizes after 70 ms.

Additionally, it is also observed that the NFNS droplets exhibit ripple formations around their maximum spreading, while NFWS droplets spread relatively smooth. Similar behavior is observed in a prior study as well [44]. This phenomenon can be attributed to the presence of SDS. The SDS, which is a kind of dispersant, reduces surface tension of the liquid. Upon impacting the wall, NFWS droplets form a smoother surface more readily due to the reduced surface tension. The surface tension counteracts irregularities around the droplet perimeter. Thus, the formation of ripples will be reduced. In contrast, NFNS droplets may exhibit uneven surfaces due to higher surface tension, leading to ripple formation as they spread and stabilize. Thus, it is evident that the presence of SDS reduces surface tension, resulting in a smoother droplet surface and potentially eliminating ripples formation around the droplet.

**Figure 5:**
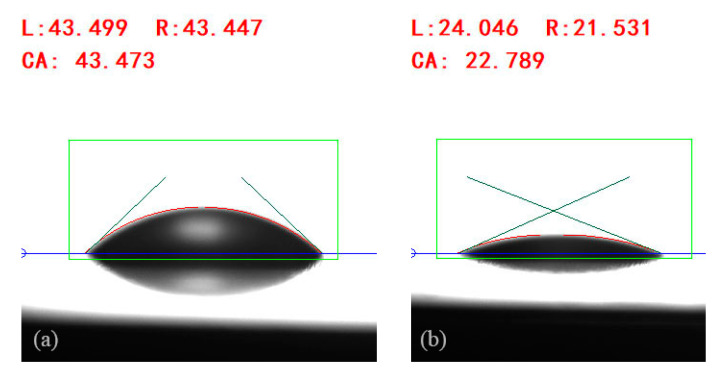
The contact angles of the NFNS (**a**) and NFWS (**b**) droplets contacting the aluminum plate.

The contact angle of nanofluid droplets at 0.5% concentration (NFNS and NFWS) are about $43.473^{\circ }$ and $22.789^{\circ }$, respectively, as shown in Figure 5. All contact angle information for NFNS and NFWS concentrations is given in Table 5. Therefore, the impacting wall in present work is hydrophilic material. The splash process of the droplets is not observed in this study, primarily attributed to the hydrophilic nature of the 7050 aluminum alloy surface. The insufficient energy for droplet splash may be a result of the following two reasons: the extensive spreading of droplets and the significant energy dissipation during surface interaction.

### 3.2. Effects of Weber Number on the Impaction Process

**Figure 6:**
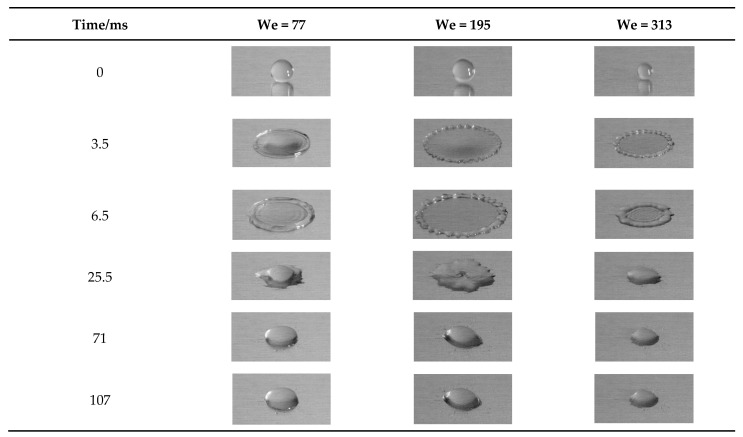
NFNS droplets impact at 0.1% concentration for different Weber numbers.

The Weber number of droplets is one of the important impacting factors. In this work, the impacted velocity ranges from 1.13 m/s to 2.28 m/s (Table 3) at an ambient temperature of 293 K. When the nanofluid concentration is 0.1%, Figure 6 shows the spreading process of NFNS droplets hitting the aluminum plate at different Weber numbers (We = 77, 195 and 313). In the initial phase of the impact of Al_2_O_3_ nanofluid droplets on the wall, the droplets spread rapidly (about 0–6.5 ms). This is due to the fact that the droplet has a large inertial potential energy at the early stage of impact, which makes the droplet spread and deform rapidly in the transverse direction under the action of inertial force.

In the initial phase, the spreading diameter of droplet increases with the time since the inertial force plays a dominant role. Subsequently (about 6.5–25.5 ms), the droplets retract. After receding to a certain extent, the droplet spreading diameter remains almost constant.

**Figure 7:**
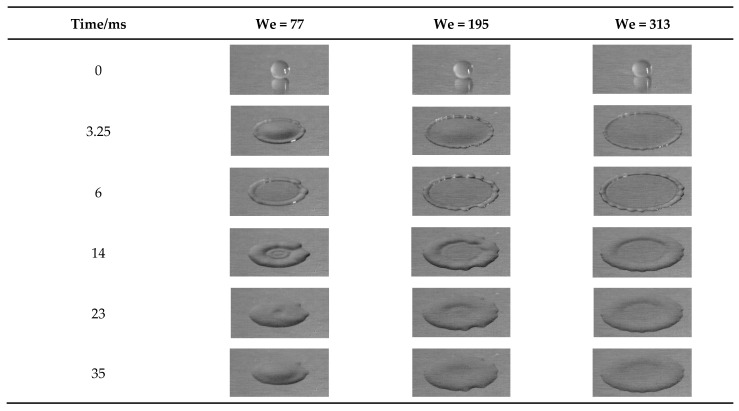
NFWS droplet impact at 0.1% concentration for different Weber numbers.

In comparison, Figure 7 shows the spreading process of NFWS droplets impacting on the wall at different Weber numbers (We = 112, 286 and 459). Initially, the NFWS droplets show the same effect. The spreading diameter is maximized by the inertial force (about 6 ms). However, it seems to have no receding phase, remaining in equilibrium (after 6 ms).

**Figure 8:**
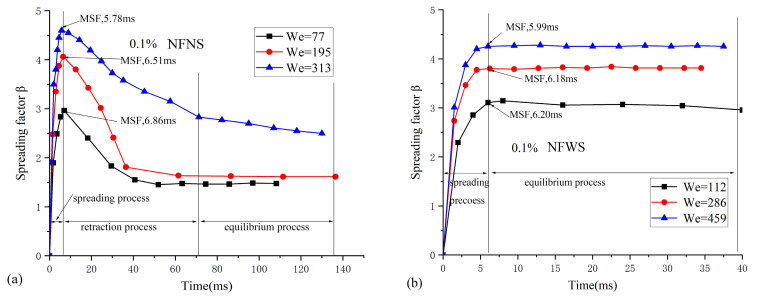
The effects of Weber number on the spreading factor at 0.1% concentration: (**a**) NFNS and (**b**) NFWS.

To evaluate quantitatively, the curves of dimensionless spreading factor with time for different Weber number at a concentration of 0.1% NFNS droplets (Figure 8a) and NFWS droplets (Figure 8b) are shown in Figure 8.

As can be seen in Figure 8, with the initial impacted velocity increasing, the kinetic energy of the impact increases, resulting in an increase in the spreading diameter. Both NFNS droplets (Figure 8a) and NFWS droplets (Figure 8b) show the same effect. For the NFNS droplets (Figure 8a), the time of maximum spreading factor (MSF) decreases with the increased Weber number. When the Weber number is 77, the time of MSF is 6.86 ms. The time of MSF are 6.51 ms and 5.78 ms for Weber numbers of 195 and 313, respectively. The receding of droplet is found for all Weber number conditions, followed by the equilibrium process after the end of the receding. The results show that the final diameter of the nanofluid droplets increases with the increase in the Weber number.

In Figure 8b, as the Weber number increases, a similar impact behavior is found before reaching the MSF. However, after the MSF, the spreading diameter remained unchanged for different Weber number. That is to say, only two processes (spreading and equilibrium) are found for the NFWS droplets to impact behaviors.

### 3.3. Effects of Surfactant and Fluid Concentration on the Impaction Process

**Figure 9:**
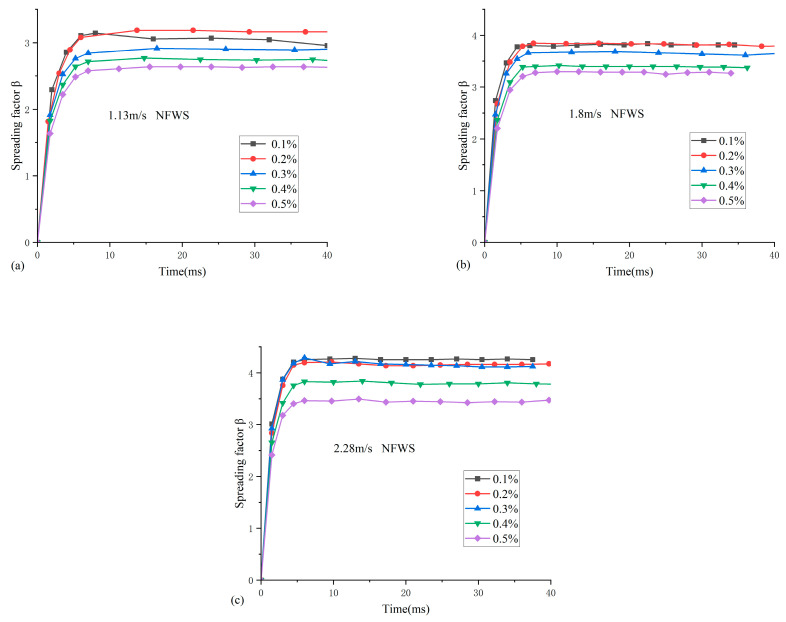
The spreading factor curves of nanofluidic droplets at different impact velocities of NFWS: (**a**) 1.13 m/s, (**b**) 1.8 m/s vs. (**c**) 2.28 m/s.

**Figure 10:**
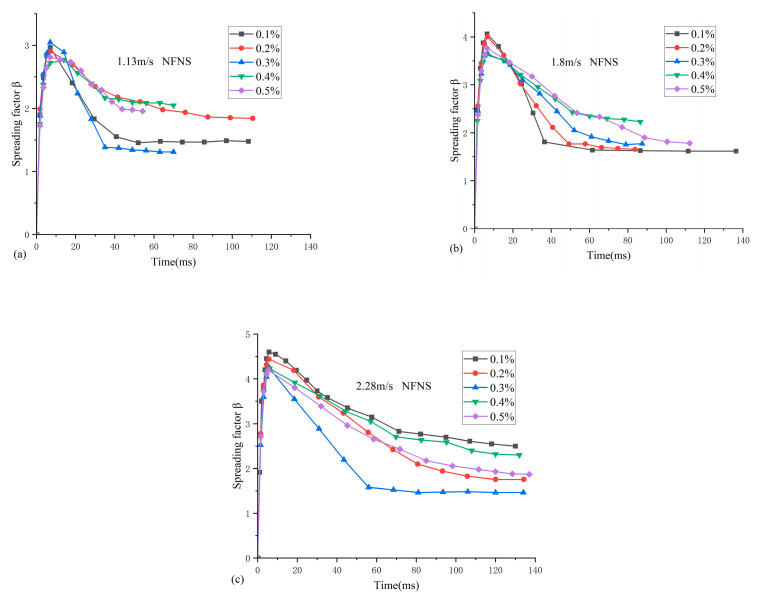
The spreading factor curves of nanofluidic droplets at different impact velocities of NFNS: (**a**) 1.13 m/s, (**b**) 1.8 m/s vs. (**c**) 2.28 m/s.

Considering the addition of SDS for nanofluids, curves of dimensionless spreading factor with time for different concentrations and impact velocities are shown in Figure 9. And as a comparison, the curves of the dimensionless spreading factor without the addition of SDS are shown in Figure 10. For the NFWS droplets (Figure 9), the droplets enter the equilibrium process directly when the spreading reaches the maximum. In contrast, for the NFNS droplets (Figure 10), the droplets show a receding process after reaching the MSF. Finally, the droplets enter the equilibrium process.

The impacting processes of NFNS and NFWS droplets exhibit significant differences, which are related to the physical parameters of the fluid, e.g., surface tension. The physical parameter, together with the impact energy from the impact velocity, determine the subsequent morphology of the impacting processes. In present work, the surface tension of NFWS droplets is smaller than that of NFNS droplets.

In Figure 9, it is evident that with the concentration of the nanofluid increasing, the spreading process is inhibited. This is because as the concentration of nanoparticles increases, the surface tension and viscosity of the nanofluid also increase. The increased surface tension and viscosity inhibits the spreading of nanoparticles on the dispersions surface, resulting in a decrease in the MSF and the final spreading factor.

In Figure 10, there is no appreciable change in the impacting process with concentration increase. When the impacting velocity is 1.13 m/s, the minimum spreading diameter is obtained at concentration of 0.3%. A similar trend is found at an impacting velocity of 2.28 m/s. However, at an impacting velocity of 1.8 m/s, the minimum spreading diameter is obtained at a concentration of 0.1%.

### 3.4. Empirical Models of the MSF

When a droplet impacts on an aluminum plate, it spreads across the plate. During the spreading process, the MSF is reached. The MSF of a droplet is commonly defined as the ratio of its maximum spreading diameter to its initial diameter, denoted as $\beta_{\max}=D_{\max}/D_{0}$, where $D_{\max}$ is the maximum spreading diameter of the droplet. The MSF plays a crucial role in industrial applications, e.g., spray cooling and surface coating.

There are numerous relations for predicting the MSF by data regression, energy conservation, mass conservation, and momentum conservation. These models typically incorporate parameters such as We, Re (Re=ρVD/μ), and Oh (Oh=We1/2/Re). The maximum spreading factor of a droplet impacting on a solid surface has been reported, and a number of empirical and theoretical models have been proposed. Table 6 lists some typical empirical models for droplets impacting on solid surfaces [45,46,47,48]. It should be noted that the viscosity values are cited from reference [49]. Since the concentrations are too low, the influence of nanoparticles is insignificant [32]. Therefore, all the viscosity values are same for the NFNS and NFWS at different concentrations.

**Figure 11:**
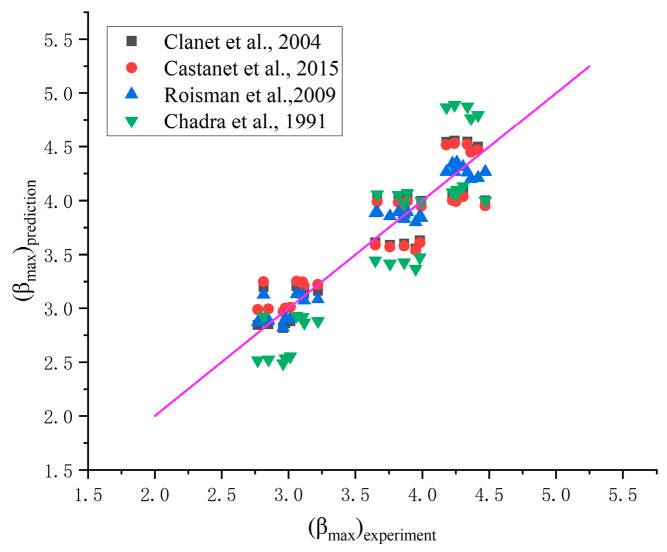
Comparison of several models from Table 6 and the measured $\beta_{\max}$ in this work [45,46,47,48].

In addition, compared with the previous model from Refs. [46,47,48], the prediction results are shown in Figure 11.

It is clear that the maximum corresponding coefficient of determination $R^{2}$ is 0.837 based on the model of Clanet et al. [45]. Stow & Hadfield [50] also described the model, $\beta_{\max}\propto We^{0.25}$, which was applied to water impacting a smooth aluminum plate. Tran et al. [51] described that the model could also applied when a droplet impacted on nonheated (hydrophilic or hydrophobic) surfaces. Thus, the model, $\beta_{\max}\propto We^{0.25}$, is more suitable for predicting the maximum spreading factor of low viscosity fluids impacting solid surface, and the largest disagreement is about 16%.

**Figure 12:**
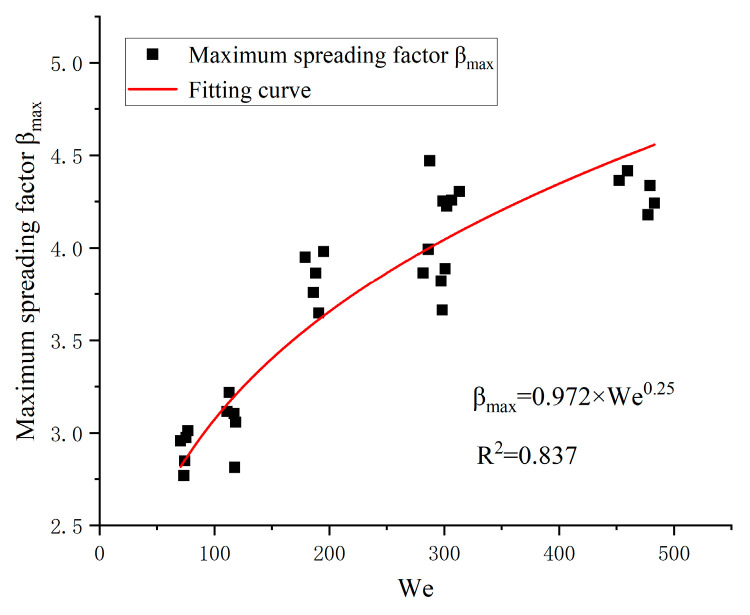
Maximum spreading factor $\beta_{\max}$ against We.

This work mainly focuses on low viscosity nanofluids impacting on solid surfaces. The impacting wall is hydrophilic material in the present work. Considering the nanofluid droplets impact for all conditions (NFNS and NFWS droplets), the correlation can be expressed as follows (Figure 12):

(8) $$\beta_{\max}=0.972We^{0.25}$$

Hence, this model can roughly predict the MSF. According to Table 2, there is little difference in the density of NFNS and NFWS droplets, whereas surface tension varies significantly. As can be seen from Equation (5), when the impact velocity, $v_{d}$, is constant, the surface tension significantly influences the MSF. Based on this finding, to enhance the predictive accuracy of the model, Equation (8) should be modified, and an improved empirical model for maximum spreading factor is proposed. The MSF of a nanofluid is predicted considering SDS addition, yielding the following correlation equations:

(9) $$\left\{\begin{array}{l} \beta_{\max}=1.026We^{0.25}\;NFNS\\ \beta_{\max}=0.929We^{0.25}\;NFWS \end{array}\right.$$

**Figure 13:**
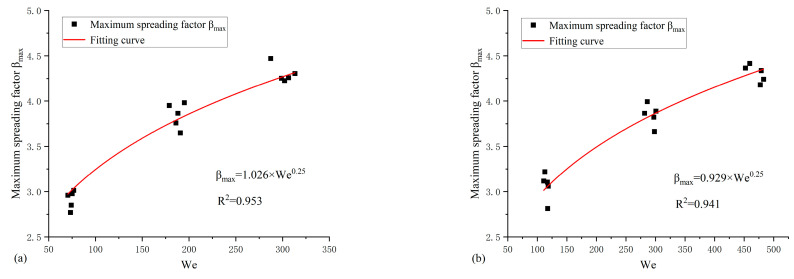
Maximum spreading factor $\beta_{\max}$ against We. (**a**) NFNS and (**b**) NFWS.

From Figure 13, it is evident that the corresponding coefficients of determination, $R^{2}$, are 0.953 and 0.941 for NFNS and NFWS droplets, respectively. This is a significant 10% improvement over the overall model. This improvement improves the accuracy of the model. The range of We values considered here is 70.481 to 482.978. The coefficients of the empirical equations of NFNS and NFWS droplets are different, which is mainly attributed to their different surface tension.

**Figure 14:**
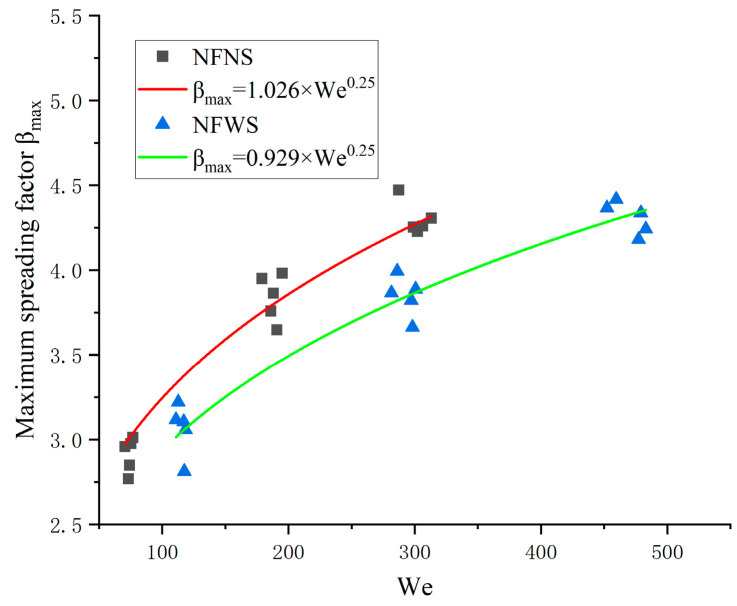
The fitting curve of the maximum spreading factor $\beta_{\max}$ and We.

It can be seen from Figure 14 that the $\beta_{\max}$ of NFNS droplets is higher than that of NFWS droplets when using the same We number.

The fluid properties are modified by the nanoparticles and SDS. According to the equation of $We=\frac{\rho_{f}v_{d}^{2}D_{0}}{\sigma }$, if the liquid density and droplet diameters are set as unchanged, the surface tension will show a positive correlation with impact velocity in the case of same We number. In the present work, the NFNS and NFWS have a similar liquid density and droplet diameter (Table 2 and Table 3). And the surface tension of NFNS is larger than that of NFWS. Therefore, at the same We number, the NFNS droplets will have higher impact velocity and momentum, resulting in a larger spreading diameter for the NFNS droplets. Consequently, the coefficient of NFNS droplets is larger than that of NFWS droplets. The physical mean of the coefficient shows the spreading capacity. Similar behavior is observed in prior studies as well [52,53,54]. In the study of Lee et al., it was also pointed out that the main driving energy for diffusion on the surface of a droplet was the initial kinetic energy, which was later covered into surface energy. In the earlier time of impacting, the kinetic energy primarily promoted the spreading at the rim [55].

In addition, the current investigation is confined to the case of room temperature. Further studies are required to understand the kinetic effects of NFNS and NFWS droplets hitting the wall at high temperature conditions. Future studies will provide a more theoretical basis and specific guidance for the thermal management experimental characterization of UAV electronic devices.

## 4. Conclusions

In the present study, Al_2_O_3_ nanofluids with different concentrations are configured by the two-step method. Single droplet impact experiments are carried out on the aluminum plate. Subsequently, the surface tension of the nanofluid is measured. The impact behaviors of nanofluid droplets on the wall are analyzed based on the high-speed imaging technology, considering concentrations, Weber number, and the absence/presence of a dispersant. The primary conclusions are as follows:

(1) The surface tension value of NFNS droplets is slightly higher than the value of DI water. The surface tension value of NFNS droplets increases with the increase in nanofluid concentration. The surface tension value of NFWS droplets is about 37.5 mN/m, which is about a 50% reduction in surface tension compared to DI water. And the change in the concentration of the nanofluid does not have much effect on the surface tension value.
(2) The impacting behaviors of all Al_2_O_3_ nanofluids are strongly affected by the Weber number. For all fluids, the MSF and the final spreading diameter in equilibrium process increase with the increase in Weber number.
(3) The NFWS droplets show spreading and equilibrium processes at different concentrations and Weber numbers, but no receding process appeared. Furthermore, as the concentration of the nanofluid increases, the MSF is inhibited. In contrast, the NFNS droplets display spreading, receding, and equilibrium processes at different concentrations and Weber numbers. And the impact of the droplets does not undergo appreciable change with increasing concentration.
(4) Considering both NFNS and NFWS droplets, the empirical equation can roughly predict the MSF. And the largest disagreement is about 16%. To improve the prediction accuracy, the empirical equations are established considering the NFNS and NFWS droplets, respectively. The corresponding coefficient of determination is significantly higher by 10% compared to the overall model, showing good agreement with the experimental results.

## Figures and Tables

**Table 1:** Main parameters of experimental materials used for nanofluid preparation.

Experimental Material	Molar Mass	Densities/g·cm^−3^	Purity	Manufacturer
Al_2_O_3_	101.96	3.5	99.9%	XFNANO
DI water	18.02	1	99%	-
SDS	288.38	1.03	99%	SINOPHARMS

**Table 2:** The nanofluid parameters.

Concentration (Vol.%)	Density/g·cm^−3^ (NFNS)	Density/g·cm^−3^ (NFWS)	Surface Tension (NFNS)	Surface Tension (NFWS)
0.1	1.002500	1.002575	63.84	37.28
0.2	1.005000	1.005075	71.03	37.29
0.3	1.007500	1.007575	71.74	37.58
0.4	1.010000	1.010075	72.16	37.82
0.5	1.012500	1.012575	72.58	38.12

**Table 3:** The impact conditions for nanofluid droplets. (A: NFNS; B: NFWS).

Parameters	0.1%		0.2%		0.3%		0.4%		0.5%	
	A	B	A	B	A	B	A	B	A	B
Droplet diameter (mm)	3.84	3.29	3.91	3.45	4.20	3.44	4.11	3.26	4.17	3.46
Droplet velocity (m/s)	1.13									
	1.80									
	2.28									

**Table 4:** Summary of errors.

Sources of Error	Error
Height of dropper	±0.1 mm
Initial diameter of droplet	±0.2 mm
Weight of surfactant	±0.1 g
Weight of nanoparticles	±0.1 g
Surface tension	±0.1 mN/m
Contact angle	$\pm 1^{\circ }$
$D_{0}$	±2.5%
$\beta_{\max}$	±4.1%
Weber number	±7.38%

**Table 5:** Contact angles of NFNS and NFWS.

Concentrations	NFNS	NFWS
0.1%	72.859	54.852
0.2%	70.560	50.479
0.3%	62.068	48.989
0.4%	56.782	42.681
0.5%	43.473	22.789

**Table 6:** Different models for the MSF as a function of the impact parameters.

Researchers	Expressions	a, b	$R^{2}$
Clanet et al. [45]	$\beta_{\max}=aWe^{0.25}$	a = 0.972	0.837
Castanet et al. [46]	$\beta_{\max}=aWe^{1/2}+b$	a = 0.17705	0.56361
Roisman [47]	βmax=aRe0.2−bRe0.4We	a = 0.976, b = 0.6922	0.83153
Chadra et al. [48]	βmax=a(Re2Oh)1/6	a = 0.60497	0.78852

## Data Availability

No new data were created nor analyzed in this study. Data sharing is not applicable to this article.
